# Structural and biochemical analysis of the IBV nsp15 endoribonuclease reveals the necessity of peripheral site residues for activity

**DOI:** 10.64898/2026.08.28.747898

**Published:** 2026-08-31

**Authors:** Ena S. Tully, Robert N. Kirchdoerfer

## Abstract

Infectious bronchitis virus (IBV) is a member of the *Gammacoronavirus* genus responsible for respiratory illness and weakened eggshells in infected chickens, adversely impacting the poultry industry. Escaping innate immune detection during infection is crucial for coronavirus proliferation in the host. The production of double-stranded RNA during coronavirus replication triggers innate immune sensors to create an antiviral state within infected cells. To counter this response, coronaviruses employ nonstructural protein 15 (nsp15) endoribonuclease to degrade double-stranded RNA. Here, we use cryo-electron microscopy and biochemistry to characterize IBV nsp15’s interactions with RNA. While the overall structure and active site of IBV nsp15 strongly resemble previous studies of nsp15 from other coronaviral genera, we note that double-stranded RNA contacts several non-conserved residues peripheral to the enzyme active site. Our data show that these residue positions can have strong impacts on RNA cleavage suggesting unique solutions for RNA engagement across coronavirus species. We also demonstrate a preference for IBV nsp15 to cleave double-stranded RNA over single-stranded RNA and observe nsp15 hexamers with two double-stranded RNAs bound simultaneously. This study reenforces the need to study diverse coronavirus species to identify distinct viral enzyme characteristics.

**GRAPHICAL ABSTRACT:**

## Full Text Availability

The license terms selected by the author(s) for this preprint version do not permit archiving in PMC. The full text is available from the preprint server.

